# Leber Hereditary Optic Neuropathy With Significant Visual Recovery: An MT-ND6 Mutation in a Malay Patient

**DOI:** 10.7759/cureus.71210

**Published:** 2024-10-10

**Authors:** Muhammad Arif Ozir, Mohammad Hudzaifah Nordin, Syaratul Emma Hashim, Sumaiyah Adzahar, Muhammad Aizuddin Ahmad, Kwang Sheng Ng, Wan-Hazabbah Wan Hitam

**Affiliations:** 1 Department of Ophthalmology, Hospital Sultan Zainal Abidin, Kuala Terengganu, MYS; 2 Faculty of Medicine, Universiti Sultan Zainal Abidin Medical Campus, Kuala Terengganu, MYS; 3 Department of Pathology, Hospital Sultan Zainal Abidin, Kuala Terengganu, MYS; 4 Department of Ophthalmology, Hospital Sultanah Nur Zahirah, Kuala Terengganu, MYS; 5 Department of Ophthalmology and Visual Science, School of Medical Sciences, Universiti Sains Malaysia Health Campus, Kubang Kerian, MYS

**Keywords:** idebenone, leber hereditary optic neuropathy, mitochondrial disease, optic neuritis, optic neuropathy

## Abstract

Leber hereditary optic neuropathy (LHON) is a rare maternally inherited mitochondrial disorder that predominantly affects young men, leading to optic nerve degeneration and subsequent vision loss. The rarity of LHON and its clinical similarity to optic neuritis complicates diagnosis, necessitating genetic testing to confirm specific point mutations and predict visual outcomes. We report a rare case of an 18-year-old Malay male with m.14484T>C/MT-ND6 mutation of LHON, who demonstrated remarkable spontaneous visual recovery over a three-year follow-up period. This report highlights the pivotal role of genetic testing in diagnosing LHON, explores the variability in visual outcomes associated with different mutations, and underscores the potential for spontaneous recovery in specific mutation variants. Early diagnosis, genetic counseling, and supportive management are critical for optimizing outcomes and improving quality of life.

## Introduction

Leber hereditary optic neuropathy (LHON), first described by Theodore Leber in 1871, is a rare mitochondrial disorder causing subacute bilateral vision loss due to retinal ganglion cell degeneration. It is linked to mitochondrial mutations that disrupt superoxide regulation, leading to retinal ganglion cell death and subsequent optic neuropathy [1].

The condition predominantly affects males, particularly those aged 20-30 years [2,3]. Approximately 90% of LHON cases are caused by three key mitochondrial point mutations: m.11778G>A/MT-ND4, m.3460G>A/MT-ND1, and m.14484T>C/MT-ND6, each associated with distinct visual outcomes [4]. Diagnosing LHON can be challenging due to its overlap with conditions like optic neuritis, particularly in atypical presentation in young adults. Comprehensive history-taking and a detailed physical examination are crucial for accurate diagnosis. Moreover, delayed diagnosis is always the case, often requiring several months to confirm through genetic testing, which may also involve significant financial implications [5]. Although LHON is typically inherited maternally, sporadic mutations can also occur in the absence of a family history [6].

Among these mutations, m.11778G>A/MT-ND4 and m.3460G>A/MT-ND1 are linked to the poorest visual prognosis with a visual recovery rate of only around 5% [7]. In contrast, the m.14484T>C/MT-ND6 mutation, which is commonly found among French Canadians, is associated with the most favorable visual prognosis, with up to 37% of patients experiencing visual recovery [8,9]. We present a rare case of m.14484T>C/MT-ND6 mutation variant LHON in an 18-year-old Malay male, notable for his remarkable visual recovery over a three-year follow-up period. This case underscores the importance of genetic testing in differentiating LHON from other optic neuropathies, particularly given the potential for visual recovery in patients with specific mutations.

## Case presentation

An 18-year-old Malay student, with no significant medical history, presented with progressive bilateral visual loss over two months. The condition initially affected his right eye and subsequently involved his left eye. He initially attributed them to refractive error; however, the rate of visual deterioration accelerated significantly during the last two weeks prior to his visit. The delayed presentation was due to his reliance on his left eye for clear vision until both eyes suffered a significant visual decline. It was painless, and he did not notice any specific visual field restriction nor any diplopia, increased intracranial pressure symptoms, neurological symptoms, or any associated fever. This was his first occurrence of visual loss, and he had no history of blindness running in the family. He was a non-smoker and did not consume alcohol.

**Figure 1:**
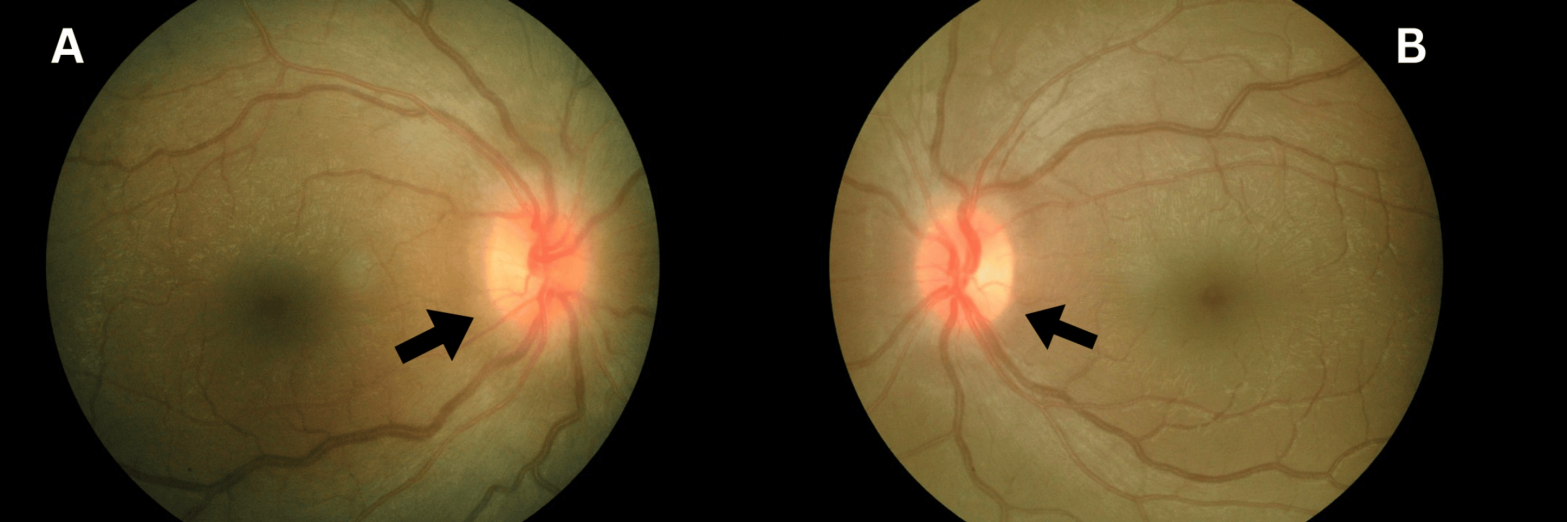
Bilateral temporal disc pallor with disc hyperemia at the nasal aspect. The margins were, however, well-defined with a normal cup-disc ratio (CDR) of 0.3 (black arrows). (A) Right eye; (B) Left eye

On initial presentation, his visual acuity was reduced to hand movement (HM) in both eyes, with no relative apparent pupillary defect (RAPD) observed. Other optic nerve function examinations revealed reduced light perception of only 60% in the right eye and 70% in the left eye. Red saturation was also diminished, with only 30% in both eyes. A confrontation test revealed a central scotoma in both eyes. Anterior segment assessment was unremarkable, with normal intraocular pressure (16 mmHg in both eyes). Posterior segment examination revealed bilateral temporal disc pallor with disc hyperemia nasally, with a normal cup-disc ratio and well-defined margins. There were no telangiectatic capillaries or retinal arterioles tortuosity, and both maculae appeared normal (Figure 1). 

**Figure 2:**
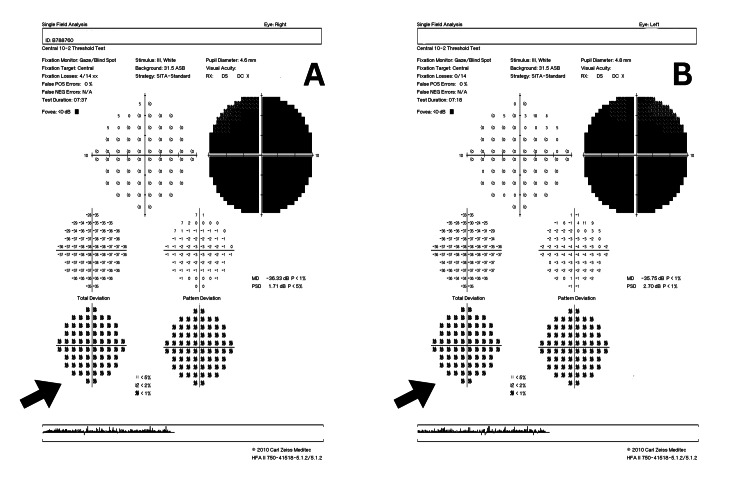
Humphrey visual test (10-2) of both eyes showing central scotoma during the first presentation (black arrows). (A) Right eye; (B) Left eye

**Figure 3:**
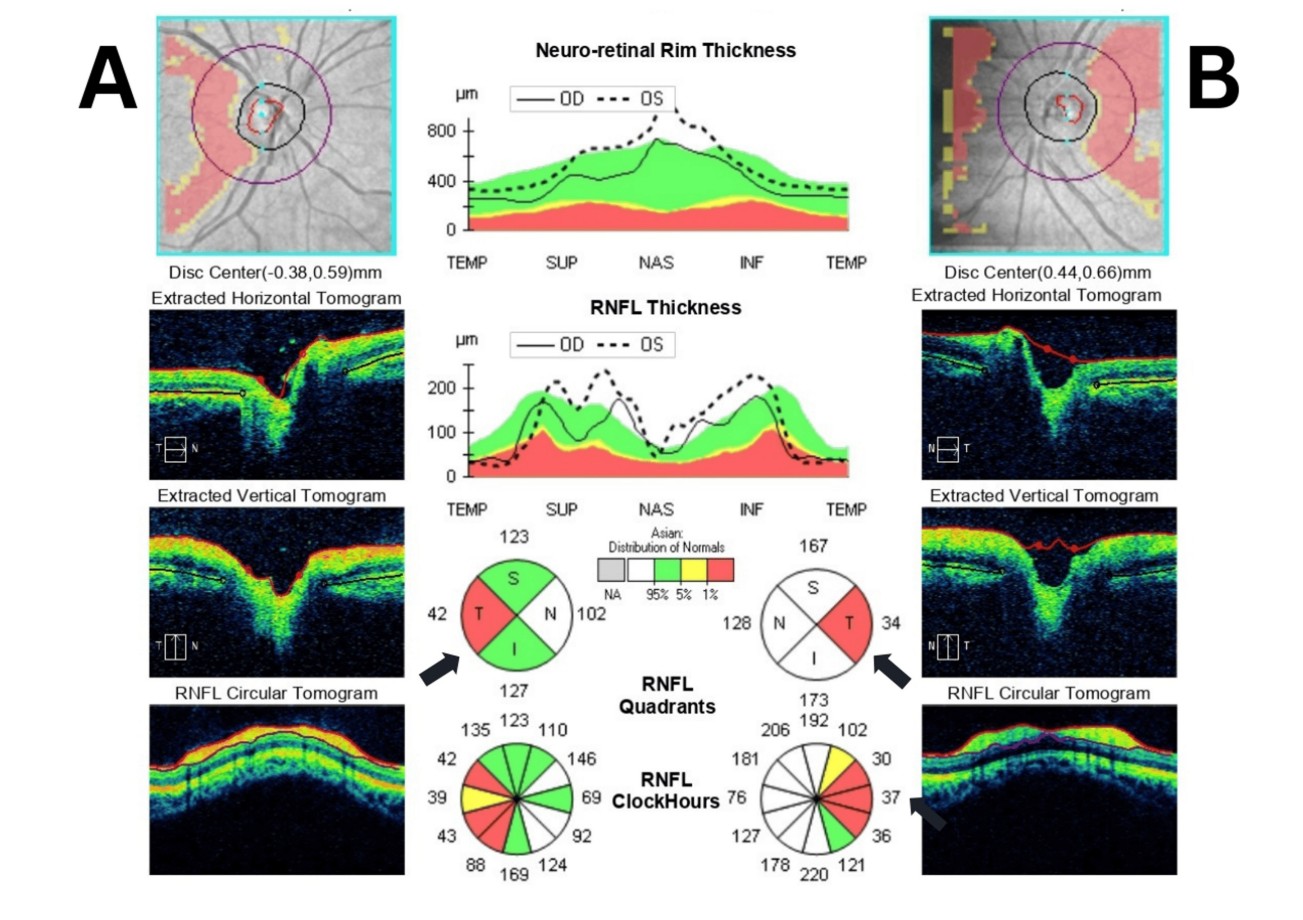
Optical coherence tomography (OCT) retinal nerve fiber layer (RNFL) of bilateral eyes shows thinning in the temporal area (black arrows). (A) Right eye; (B) Left eye

Perimetry revealed a generalized reduction in the visual field based on the central 10-2 threshold Humphrey visual field (HVF) test (Figure 2). Optical coherence tomography (OCT) of the retinal nerve fiber layer (RNFL) in both eyes showed thinning in the temporal quadrant (Figure 3). A visual evoked potentials (VEP) study indicated impaired bilateral optic pathway function. Additionally, his blood workup of anti-aquaporin 4 antibody and myelin oligodendrocyte glycoprotein (MOG) antibody tests were negative, ruling out demyelinating causes. Screening for connective tissue disease was also negative. Metabolic workups such as vitamin B12 and folate were negative. Magnetic resonance imaging (MRI) of the brain and orbits was normal with both optic nerves appearing normal and symmetrical. 

In the initial part, he was diagnosed with bilateral optic neuritis and treated with intravenous methylprednisolone at a dose of 250 mg four times daily for three days, followed by oral prednisolone 1 mg/kg for 11 days and then tapered off over five weeks. We observed no clinical improvement during the two-week course of treatment. 

During the first six months of the follow-up period, his clinical condition remained unchanged with the vision remaining static with hand movement for both eyes. After further discussions and counseling, his parents consented to a genetic study to confirm or rule out LHON. The blood results of the study were positive for the MT-ND6 gene, revealing a DNA mutation of m.14484T>C/MT-ND6 with 40% heteroplasmy, which led to a revised diagnosis of LHON (Table 1).

**Table 1:** Leber hereditary optic neuropathy (LHON) genetic test. Mitochondrial DNA (mtDNA) pathogenic variant of the MT-ND6 gene with a DNA change of m.14484T>C.

Gene (RefSeq/LRG)	DNA Change	Protein Change	% Heteroplasmy	Classification
MT-ND6 (NC_012920.1)	m.14484T>C	p.(Met64Val)	Approximately 40%	Pathogenic

**Figure 4:**
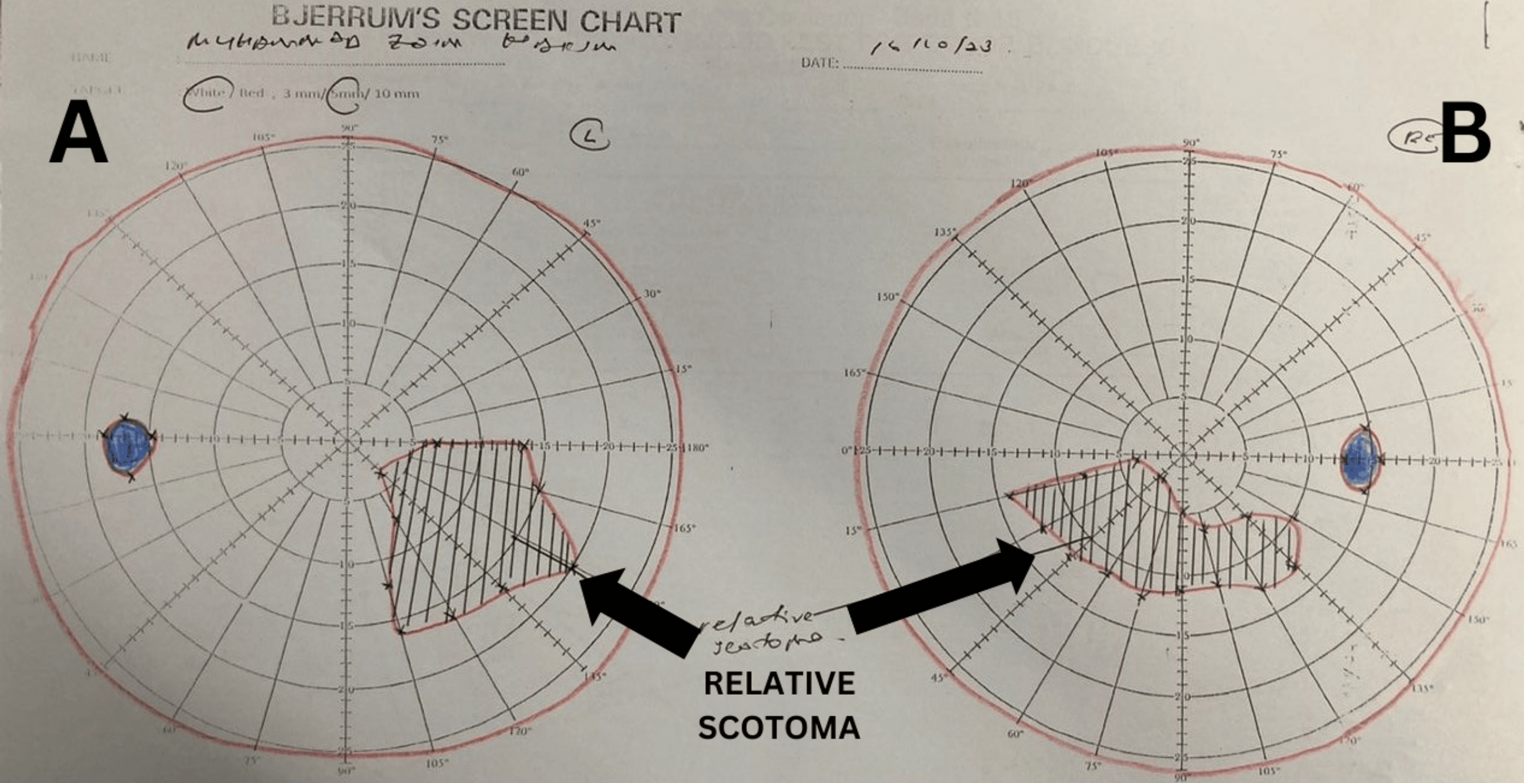
Bjerrum chart of both eyes showing residual small degree of scotoma in both eyes during the latest visit (black arrows). (A) Left eye; (B) Right eye

After one year of follow-up, his vision improved to 6/60. Due to his condition, he missed over a year of schooling. With continued four-monthly reviews, his vision steadily improved, and by the three-and-a-half-year mark, his vision had fully recovered to 6/6 in both eyes. His Bjerrum chart showed gradual recovery, with a small degree of scotoma remaining in his central vision at his most recent follow-up (Figure 4). Notably, he achieved remarkable visual recovery and remains stable with no recurrences. He is currently well, on regular six-monthly follow-ups, and successfully pursuing his studies at the tertiary level.

## Discussion

LHON is a rare mitochondrial disorder caused by mutations in mitochondrial DNA (mtDNA), disrupting the electron transport chain, particularly at the subunit encoding complex I, which is the first enzyme in the respiratory chain responsible for mitochondrial energy production. The prevalence of LHON is approximately one in 31,000 in the Northern UK and one in 54,000 in Denmark [10], with limited epidemiological data from Southeast Asia regions. To our knowledge, the only available number comes from Thailand, where a total of 20 cases of m.14484T>C/MT-ND6 mutation were diagnosed over a 20-year review period [11]. Among the three primary mutations, m.11778G>A/MT-ND4 is the most common, accounting for 79% of cases, followed by m.3460G>A/MT-ND1 (17%) and m.14484T>C/MT-ND6 (4%) [12]. The m.11778G>A/MT-ND4 mutation is associated with the poorest visual prognosis, with only 5% of affected individuals experiencing visual recovery [7]. These mutations involve a point mutation that leads to the substitution of a single amino acid, resulting in a significant reduction in neuronal energy production and subsequent death of retinal ganglion cells [13].

Given its rarity, LHON imposes diagnostic challenges on clinicians and is often misdiagnosed as it shares clinical features with more common conditions affecting young adults, such as optic neuritis, especially the atypical variant. The classical presentation typically occurs in the second decade of life characterized by painless blurry vision that is usually bilateral. A positive family history of visual loss may raise clinical suspicions [2,3]. The presenting central vision loss, in this case, occurred possibly due to the involvement of a subset of macular retinal ganglion cells, which are responsible for central vision, leading to large central scotomas and temporal pallor of the optic disc [14]. Although it usually occurs bilaterally, it is asynchronous, which means the second eye is usually affected within a year. Vision loss generally stabilizes after six to 12 months after the first onset. Our patient presented late to us but with a similar clinical picture. Further questioning revealed no significant visual loss running in the family. This is possible as there are as many as 40% of patients who do not have a family history of LHON but present with the disease. Moreover, the mother of a proband usually has the mtDNA pathogenic variant and may or may not have developed visual loss. This is due to the de novo mutation being rare and family tracing can be difficult [15,16].

In early presentations, 20% of patients may show normal ocular findings [17]. Other characteristic findings, such as vessel tortuosity and telangiectatic capillaries, were not present in this case [18]. Our patient presented two months after the onset of symptoms with bilateral disc temporal pallor, suggesting that his condition may already be in the intermediate stage of LHON. Generally, LHON progresses from temporal optic nerve to diffuse optic nerve atrophy in the later stages of the disease [17]. The visual field findings for typical LHON findings are bilateral cecocentral scotomas in most cases.

To aid in the diagnosis, OCT of the RNFL is essential to detect RNFL thinning, while visual field tests classically reveal central scotomas. These, however, are unspecific tests, which may be more useful for monitoring disease progression rather than confirming the diagnosis. Additional investigations are crucial to rule out other differential diagnoses, such as demyelinating optic neuritis, infective optic neuritis, or compressive optic neuropathies. In this patient, serum anti-aquaporin 4 antibody and MOG antibody tests were negative, with inconclusive infective screening workups similar to our case. Positive VEP reports also confirmed optic neuropathy, ruling out malingering that may occur in this age group. Although it has been reported that an MRI of the brain within the first year of LHON onset may show chiasmal enlargement and T2 hyperintensity in the posterior optic nerve, these findings were not present in our case as the initial imaging study was normal [19]. A repeat MRI was not performed due to the patient's financial constraints.

It can take months to reach a confirmed diagnosis of LHON, which is ultimately confirmed through genetic testing [5]. Genetic testing is the most crucial investigation for confirming the diagnosis. Next-generation sequencing (NGS) of the mitochondrial genome serves as the current gold standard for molecular diagnosis of LHON as it can detect both primary mutations and autosomal recessive variants of the disorder [20]. Genetic testing is also important for identifying the specific mutations, which can help to predict the likelihood of visual recovery. However, relevant preliminary investigations should be conducted first as genetic testing is costly and has limited availability. There is a strong consensus among experts to clinically screen maternally related relatives of individuals with LHON, but it is generally not necessary to perform genetic screening for these groups. This is due to several economic and ethical considerations associated with genetic testing [5]. In our case, after a lengthy family counseling, the parents ultimately agreed to proceed with the test for the patient alone.

The m.3460G>A/MT-ND1 mutation variant is often associated with severe mitochondrial diseases, such as LHON and mitochondrial encephalopathy, lactic acidosis, and stroke-like episodes (MELAS) syndrome [21]. This is because MT-ND1 is located at the core of mitochondrial complex I and is essential for its structural integrity and function. Mutations in MT-ND1 can cause significant defects in mitochondrial oxidative phosphorylation, leading to more severe clinical manifestations.

MT-ND6 mutations can also be pathogenic and are associated with conditions like LHON and mitochondrial myopathy [21]. However, they are sometimes considered to have a less severe impact compared to MT-ND1 mutations because MT-ND6 is located at the periphery of mitochondrial complex I. While mutations in MT-ND6 can disrupt electron transfer and affect complex I activity, they may not destabilize the entire complex as significantly as MT-ND1 and MT-ND4 mutations do. In certain environments or under specific conditions, MT-ND6 mutations might confer a selective advantage. For instance, some studies have suggested that specific MT-ND6 mutations might resist oxidative stress or help in adaptation to hypoxic conditions. This is in contrast to MT-ND1 mutations, which tend to be more universally detrimental due to their critical role in complex I function as discussed above [21,22].

In this case, the patient has a genetic mutation identified as m.14484T>C/MT-ND6, which is associated with a favorable prognosis, particularly in younger patients. The lower degree of heteroplasmy, which was 40%, also contributed to his positive visual outcome as it is known that heteroplasmic mutation load (% of mutated mtDNA) affects disease expression. Furthermore, this mutation variant has been reported to have the best visual recovery outcomes compared to other LHON mutations as nearly 37% of patients experience significant visual recovery after a prolonged period of visual impairment [8]. The average recovery interval is typically 12 to 21 months after the onset of visual loss [8]. Johns et al. reported bilateral visual recovery in two out of 19 patients with visual acuity 6/9 in both eyes. Additionally, three out of 19 patients achieved 6/7.5 vision or better in both eyes [8]. In our case, the patient demonstrated remarkable visual recovery, achieving a final visual acuity of 6/6, although small residual visual field limitation persisted.

Idebenone is a disease-specific treatment for LHON. Idebenone functions by bypassing the disrupted complex I and directly transferring electrons to the mitochondria, thereby increasing energy supply and aiding in the restoration of inactive but viable retinal ganglion cells, which facilitate vision restoration. Studies have shown that 80% of patients treated with idebenone, compared to a placebo group, experienced an improvement of up to 0.2 based on the logarithm of the minimum angle of resolution (logMAR) chart [23,24]. Idebenone also has been effective in stabilizing and restoring vision of patients treated within one year of vision loss onset. In this case, idebenone was not an option due to its unavailability, and it has not yet received Food and Drug Administration (FDA) approval. However, it is widely used in European countries due to its preferable outcomes [25].

Other therapeutic strategies currently under investigation include gene therapy, gene editing, antioxidants, neurotrophic agents, mitochondrial biogenesis, mitochondrial replacement, and stem cell therapies. Allotopic gene therapies are the most advanced in development (currently in phase III clinical trials), while most other approaches are in phase I or II trials or at pre-clinical stages. Despite ongoing research, a definitive effective treatment for LHON is yet to be established [26].

Our patient achieved near-complete visual recovery without any specific intervention. His most recent follow-up revealed only residual scotomas on the Bjerrum chart. During the course of follow-up, he was referred to occupational therapy and provided with low vision aids, both of which have been shown to improve the quality of life [27]. Nearly three years post-diagnosis, he remains in good health with no recurrence and has been able to continue his studies at the tertiary level.

## Conclusions

This case highlights the management and diagnostic challenges associated with LHON, a rare mitochondrial disorder that is often misdiagnosed due to its overlap with other more common conditions, such as optic neuritis in young adults. It also underscores the importance of comprehensive evaluation, including genetic testing, which remains the gold standard for confirming LHON, especially in atypical cases or cases without a family history. Our patient's near-complete visual recovery suggests that individuals with the m.14484T>C/MT-ND6 mutation may achieve favorable outcomes, especially with relatively low degree heteroplasmy. Further research is needed to better understand the factors influencing visual prognosis in LHON, particularly the role of genetic mutations and potential modifiers that may impact disease progression and recovery.
